# Vector role and human biting activity of Anophelinae mosquitoes in different landscapes in the Brazilian Amazon

**DOI:** 10.1186/s13071-021-04725-2

**Published:** 2021-05-06

**Authors:** Tatiane M. P. Oliveira, Gabriel Z. Laporta, Eduardo S. Bergo, Leonardo Suveges Moreira Chaves, José Leopoldo F. Antunes, Sara A. Bickersmith, Jan E. Conn, Eduardo Massad, Maria Anice Mureb Sallum

**Affiliations:** 1 Departamento de Epidemiologia, Faculdade de Saúde Pública, Universidade de São Paulo, Av. Dr. Arnaldo, 715, Cerqueira César, São Paulo, SP 01246-904 Brazil; 2 Setor de Pós-Graduação, Pesquisa e Inovação, Centro Universitário Saúde ABC (FMABC), Fundação ABC, Santo André, SP Brazil; 3 Superintendencia de Controle de Endemias, Secretaria de Estado da Saúde, Araraquara, SP Brazil; 4 Wadsworth Center, New York State Department of Health, Albany, NY USA; 5 Department of Biomedical Sciences, School of Public Health, State University of New York, Albany, NY USA; 6 Escola de Matemática Aplicada, Fundação Getúlio Vargas, Rio de Janeiro, RJ Brazil

**Keywords:** *Nyssorhynchus darlingi*, *Nyssorhynchus rangeli*, *Nyssorhynchus benarrochi* B, *Nyssorhynchus konderi* B, *Plasmodium* vectors, Deforestation, Amazonian settlements

## Abstract

**Background:**

Environmental disturbance, deforestation and socioeconomic factors all affect malaria incidence in tropical and subtropical endemic areas. Deforestation is the major driver of habitat loss and fragmentation, which frequently leads to shifts in the composition, abundance and spatial distribution of vector species. The goals of the present study were to: (i) identify anophelines found naturally infected with *Plasmodium*; (ii) measure the effects of landscape on the number of *Nyssorhynchus darlingi*, presence of *Plasmodium*-infected Anophelinae, human biting rate (HBR) and malaria cases; and (iii) determine the frequency and peak biting time of *Plasmodium*-infected mosquitoes and *Ny. darlingi.*

**Methods:**

Anopheline mosquitoes were collected in peridomestic and forest edge habitats in seven municipalities in four Amazon Brazilian states. Females were identified to species and tested for *Plasmodium* by real-time PCR. Negative binomial regression was used to measure any association between deforestation and number of *Ny. darlingi*, number of *Plasmodium*-infected Anophelinae, HBR and malaria. Peak biting time of *Ny. darlingi* and *Plasmodium*-infected Anophelinae were determined in the 12-h collections. Binomial logistic regression measured the association between presence of *Plasmodium*-infected Anophelinae and landscape metrics and malaria cases.

**Results:**

Ninety-one females of *Ny. darlingi*, *Ny. rangeli*, *Ny. benarrochi* B and *Ny. konderi* B were found to be infected with *Plasmodium.* Analysis showed that the number of malaria cases and the number of *Plasmodium*-infected Anophelinae were more prevalent in sites with higher edge density and intermediate forest cover (30–70%). The distance of the drainage network to a dwelling was inversely correlated to malaria risk. The peak biting time of *Plasmodium*-infected Anophelinae was 00:00–03:00 h. The presence of *Plasmodium*-infected mosquitoes was higher in landscapes with > 13 malaria cases.

**Conclusions:**

*Nyssorhynchus darlingi*, *Ny. rangeli*, *Ny. benarrochi* B and *Ny. konderi* B can be involved in malaria transmission in rural settlements. The highest fraction of *Plasmodium*-infected Anophelinae was caught from midnight to 03:00 h. In some Amazonian localities, the highest exposure to infectious bites occurs when residents are sleeping, but transmission can occur throughout the night. Forest fragmentation favors increases in both malaria and the occurrence of *Plasmodium*-infected mosquitoes in peridomestic habitat. The use of insecticide-impregnated mosquito nets can decrease human exposure to infectious Anophelinae and malaria transmission. 
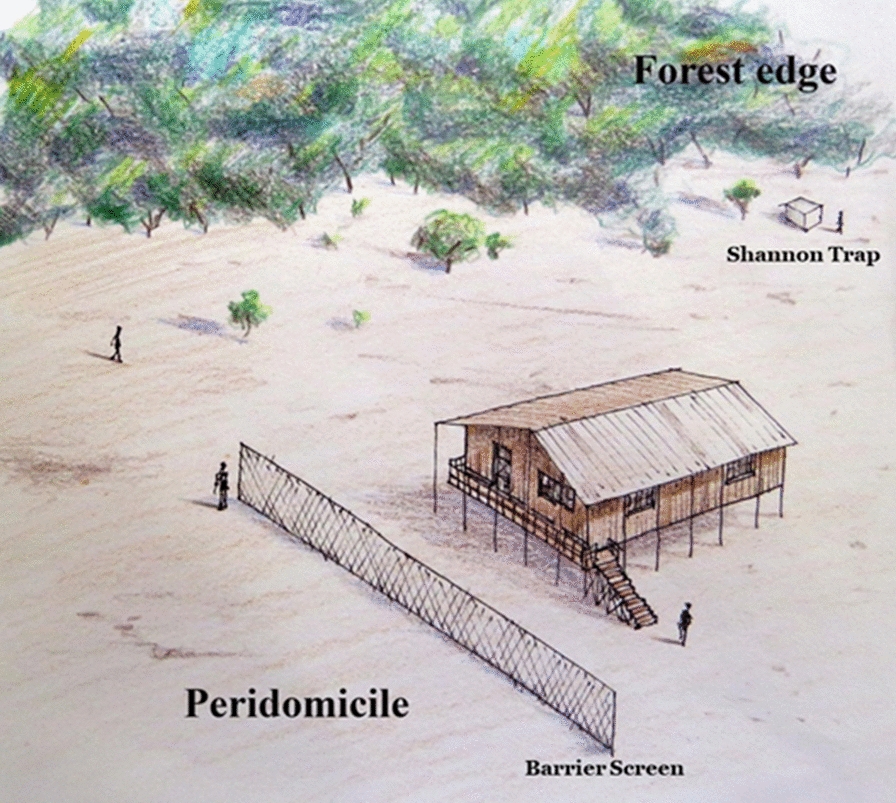

**Supplementary Information:**

The online version contains supplementary material available at 10.1186/s13071-021-04725-2.

## Background

Human malaria is mainly caused by the protozoan parasites *Plasmodium falciparum* Welch, 1897 and *P. vivax* (Grassi & Feletti, 1890). Four additional species have also been identified [1]. Approximately 70 species of the subfamily Anophelinae Grassi, 1900 are vectors of *Plasmodium* Marchiafava & Celli (1885), of which 41 are considered to be dominant vectors [2, 3]. In Brazil, *Nyssorhynchus darlingi* (Root, 1926; formerly *Anopheles darlingi*), *Ny. marajoara* (Galvao & Damasceno, 1942; formerly *Anopheles marajoara*) and *Ny. aquasalis* (Curry, 1932; formerly *Anopheles aquasalis*) are the primary vectors of human malaria [4, 5].

Despite major progress in the global control and elimination of malaria, the World Health Organization reports show alarming increases in the incidence of malaria from 2014 to 2018. In Brazil, after a 6-year period of successive reduction, the incidence of malaria increased from 117,832 cases in 2016 to 174,522 in 2017 [6]. A reduction of 19% in malaria incidence was reported from 2018 (193,837) to 2019 (156,918) [7]. Most cases (99.5%) occurred in the Amazon, with > 90% of infections caused by *P. vivax*. Malaria hotspots occurr in the Brazilian Amazon where both *P. vivax* and *P. falciparum* transmission is continuously high [8]. Such hotspots represent a challenge in terms of achieving the control and elimination of *Plasmodium* infection [9]. Some of these hotspots are found in the westernmost municipalities of Cruzeiro do Sul and Mâncio Lima (Acre state), where the proportion of malaria cases due to *P*. *falciparum* and *P*. *vivax* + *P*. *falciparum* can be as high as 25% of the overall estimated cases in Acre [7].

Environmental factors, including temperature, precipitation and humidity, can affect the risk of malaria infection [10–15]. Minor variations in temperature can increase or decrease the extrinsic incubation period of *Plasmodium*, which in turn affects the vector competence and vectorial capacity of populations of a vector species [16]. In addition, increased density of mosquito habitats in human-dominated landscapes can lead to augmented abundance of mosquito vectors, resulting in increased malaria incidence. An interrelated chain of ecological events has been shown to lead to alterations in mosquito species composition in environments that are impacted by habitat loss and fragmentation associated with anthropogenic activities [17, 18]. An increased abundance of generalist and opportunistic species [19], including vectors and infectious pathogens [20], has been shown to have a potential effect on mosquito communities. Also, changes in the spatiotemporal distribution of larval habitats can influence vector abundance and human–mosquito contact rate [21, 22]. Furthermore, changes in rural infrastructure that alter standing freshwater distribution, such as the construction of dams, reservoirs and irrigation networks, may increase malaria incidence unless they are coordinated with measures to minimize the risk of disease outbreaks [23]. In a study aimed to understand factors affecting the increase and decrease of malaria risk and the dynamics of transmission in areas that are currently experiencing ecological change and human occupation, Baeza et al. [24] demonstrated that the ecological changes that promote increases in mosquito abundance and *Plasmodium* dispersion occur at a faster rate than the socioeconomic factors that can prevent malaria transmission.

In the Amazon, deforestation is the major driver of changes in the forest landscape and also a major driver of increased malaria incidence [25]. However, the relationship between landscape change and malaria is complex and bidirectional, i.e. increased incidence of the disease in the early stages of deforestation can lead to a decrease in forest clearing [26]. Recently, Souza et al. [8] showed that the spatial pattern of malaria transmission linked to the economic expansion is primarily associated with extractive activities, human movement and agricultural settlements. In rural settlements, particularly those located in areas neighboring the forest fringe, environmental changes can increase the number of partially shaded larval habitats, thus favoring increased *Ny. darlingi* abundance [27]. In addition, socioeconomic factors impact malaria because poor housing, poor management of the environment and inadequate sanitary conditions facilitate human–mosquito contact and, thereby, can increase the human–mosquito contact rate and malaria transmission [28–30].

The dynamics of malaria are influenced by several factors linked to vector ecology, *Plasmodium* infection rate in both mosquito and human populations, human behavior and the environment. Malaria transmission in rural settlements affects the socioeconomic development, human well-being, quality of life and health of the communities, and is a major indicator of inequalities [31]. Consequently, field entomology investigations are of utmost importance in the search for an understanding of the factors that are permissive to the circulation of the infection and that challenge the success of control programs. The aim of this study was to assess how *Ny. darlingi* and other Anophelinae species contribute to malaria transmission in rural settlements in different forest clearing and fragmentation settings. Entomological field data and data on local malaria cases and landscape components were used to achieve the following aims: (i) identification of *Plasmodium* infection in anopheline species; (ii) measurement of the effects of landscape on the number of local malaria cases, the number of *Ny. darlingi* in peridomestic habitats*,* human biting rate (HBR) and number of *Plasmodium*-infected Anophelinae in peridomestic habitats; and (iii) determination of the frequency and peak biting time of *Plasmodium*-infected mosquitoes and *Ny. darlingi*.

Taking into account the malaria elimination policy in Brazil, we identified additional components that can present as challenges to vector control. For example, we found strong evidence of outdoor transmission of *Plasmodium* by *Ny*. *darlingi* and other anophelines. An interesting and yet intriguing result was that we detected *P*. *falciparum* in sylvatic anopheline species at the forest edges of Acre state. One of the novelties of this study was to show that the incidence rate ratio (IRR) of *Plasmodium* was greater after midnight, when the number of bites per human from *Ny*. *darlingi* was lower, in comparison with that in the crepuscular period.

## Methods

### Study area, mosquito collections and species identification

**Fig. 1:**
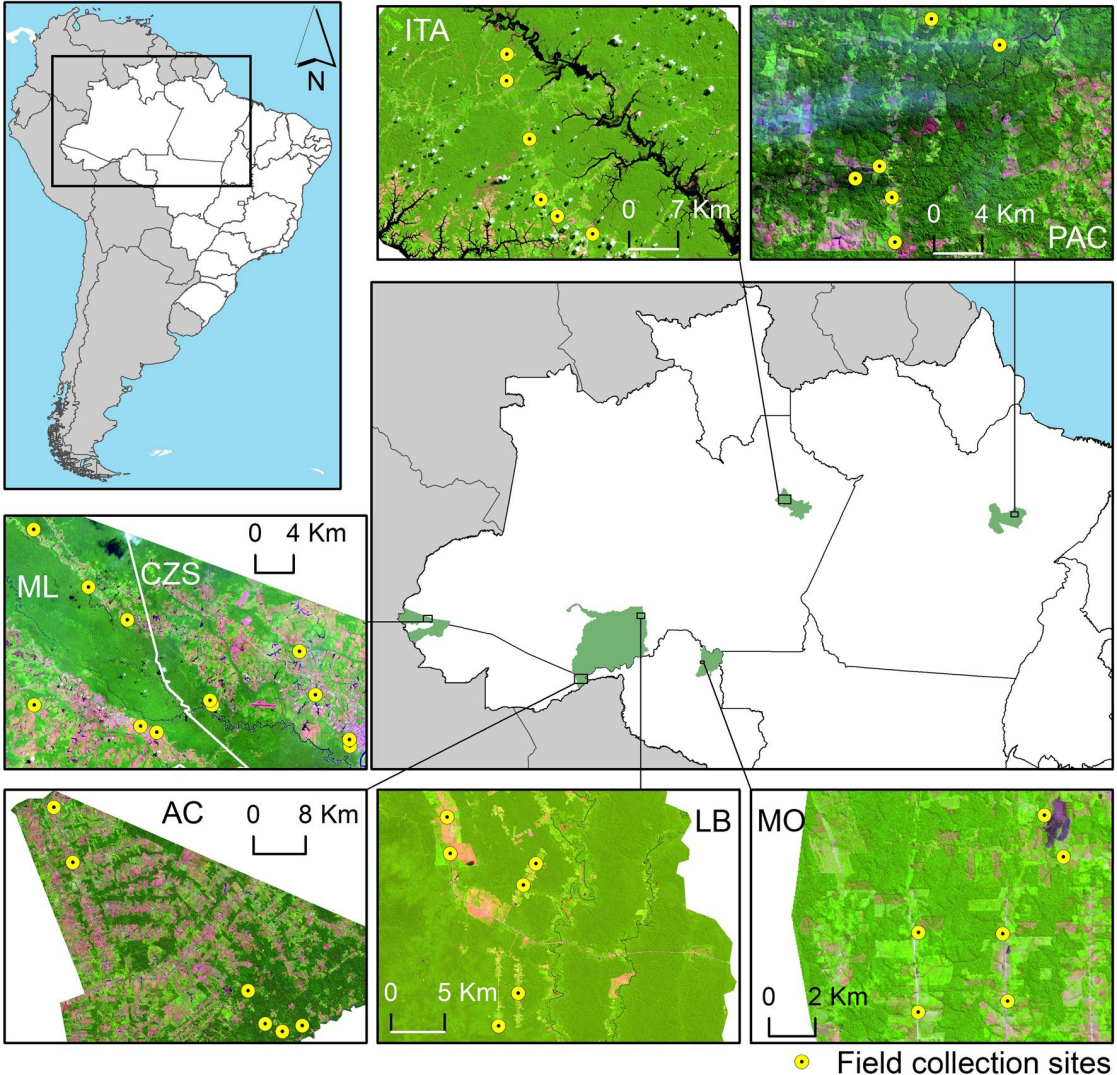
Map of the localities where the field collections were carried out.* AC* Acrelândia,* CZS* Cruzeiro do Sul,* ITA* Itacoatiara,* LB* Lábrea,* ML* Mâncio Lima,* MO* Machadinho D'Oeste, *PAC* Pacajá

Mosquitoes of the subfamily Anophelinae were collected in rural settlements in the municipalities of Acrelândia (Acre state), Cruzeiro do Sul (Acre state), Mâncio Lima (Acre state), Itacoatiara (Amazonas state), Lábrea (Amazonas state), Pacajá (Pará state) and Machadinho D'Oeste (Rondônia state), Brazil (Fig. 1). These areas are characterized by a wet season, a dry season and wet–dry transition months. The mean annual regional rainfall is > 2000 mm, and the mean temperature is approximately 26 °C. The mean annual relative humidity is approximately  59% but varies substantially with rainfall and surface water [32].

Field collections were conducted from January 2015 to November 2016. In Acrelândia, Cruzeiro do Sul and Mâncio Lima, collections were conducted from 18:00 to 06:00 h, whereas in Machadinho D’Oeste, Rondônia, Pacajá, Pará, Itacoatiara and Lábrea, collections were conducted from 18:00 to 00:00 h (Table 1). Two data sets were employed for the statistical analyses. One data set comprised the 12-h collections and the second data set included the 6-h collections at all locations. Mosquitoes were sampled outdoors for 42 houses in 23 locations. In the peridomestic habitats, mosquitoes were collected using the human landing catch (HLC) and barrier screen sampling (BSS) methods. At the forest edge, mosquitoes resting on the walls of a Shannon trap (ST) were collected [33] using both a light source and human attraction. HLC and ST collections were carried out on one night at each of the 42 houses, whereas BSS collections were conducted in peridomestic habitats of 13 houses. The selection of field localities, houses in which HLC trapping was carried out and collection protocols are described in detail in Sallum et al. [32] and Massad et al. [34]. BSS was performed by two to four collectors in each of the 13 peridomestic habitats on the day after the HLC and ST collections (Additional file 1: Figure S1). Female mosquitoes were euthanized at hourly intervals with ethyl acetate (C_4_H_8_O_2_) vapors in the field and stored in silica gel; each set was marked with the date, location, house, collection method and hour of collection. All field collections were carried out under permanent permit number 12583–1 from the Instituto Chico Mendes de Conservação da Biodiversidade (ICMBio, SISBIO) granted to one of the authors (MAMS). The collection locations were not privately owned or protected, and the field studies did not involve protected or endangered species [35].

**Table 1:** Municipality and locality collection, mosquito collection date, local malaria cases and local population

Collection point	State	ID_SIVEP^a^	Municipality	Locality	Collection date	Local malaria cases^b^	Population^c^
P1	RO	373	Machadinho D'Oeste	Linha TB 14-Galo Velho	Oct 2015	5	129
P2	RO	374	Machadinho D'Oeste	Linha TB 13-Galo Velho	Oct 2015	9	111
P3	RO	371	Machadinho D'Oeste	Linha 10-Galo Velho	Oct 2015	13	180
P4	RO	371	Machadinho D'Oeste	Linha 10-Galo Velho	Oct 2015	13	180
P5	RO	376	Machadinho D'Oeste	Linha 09-Galo Velho	Oct 2015	13	90
P6	RO	376	Machadinho D'Oeste	Linha 09-Galo Velho	Oct 2015	13	90
P1	AM	16	Lábrea	Boa Água-[P.A. Umari]	Aug 2015	33	163
P2	AM	16	Lábrea	Boa Água-[P.A. Umari]	Aug 2015	33	163
P3	AM	138	Lábrea	PA Paciá	Aug 2015	26	286
P4	AM	21	Lábrea	Apairal-[P.A. Umari]	Aug 2015	19	68
P5	AM	21	Lábrea	Apairal-[P.A. Umari]	Aug 2015	19	68
P6	AM	21	Lábrea	Apairal-[P.A. Umari]	Aug 2015	19	68
P1	AC	22	Cruzeiro do Sul	Saboeiro	April, May 2015	138	2239
P2	AC	8	Cruzeiro do Sul	Cohab	April, May 2015	58	1258
P3	AC	37	Cruzeiro do Sul	Canela Fina	April, May 2015	69	426
P4	AC	42	Cruzeiro do Sul	Igarapé Preto	April, May 2015	52	634
P5	AC	76	Cruzeiro do Sul	Humaitá	April, May 2015	37	127
P6	AC	76	Cruzeiro do Sul	Humaitá	April, May 2015	37	127
P1	AC	2	Mâncio Lima	Guarani	May, June 2015	56	1174
P2	AC	2	Mâncio Lima	Guarani	May, June 2015	56	1174
P3	AC	71	Mâncio Lima	Colônia Normando	May, June 2015	8	73
P4	AC	52	Mâncio Lima	Paraná Pentecoste	May, June 2015	189	544
P5	AC	52	Mâncio Lima	Paraná Pentecoste	May, June 2015	189	544
P6	AC	52	Mâncio Lima	Paraná Pentecoste	May, June 2015	189	544
P1	AC	110	Acrelândia	Reserva Porto Dias	Jan 2015	6	544
P2	AC	110	Acrelândia	Reserva Porto Dias	Jan 2015	6	544
P3	AC	110	Acrelândia	Reserva Porto Dias	Jan 2015	6	544
P4	AC	110	Acrelândia	Reserva Porto Dias	Jan 2015	6	544
P5	AC	127	Acrelândia	Reserva Porto Luiz	Jan 2015	0	209
P6	AC	127	Acrelândia	Reserva Porto Luiz	Jan 2015	0	209
P1	PA	332	Pacajá	Invasão (Cururuí)	April 2016	5	112
P2	PA	332	Pacajá	Invasão (Cururuí)	April 2016	5	112
P3	PA	332	Pacajá	Invasão (Cururuí)	April 2016	5	112
P4	PA	343	Pacajá	Cururuí–Núcleo G	April 2016	2	123
P5	PA	334	Pacajá	Cururuí–Núcleo F	April 2016	3	189
P6	PA	332	Pacajá	Invasão (Cururuí)	April 2016	5	112
P1	AM	375	Itacoatiara	Ramal do Incra	Nov 2016	1	106
P2	AM	375	Itacoatiara	Ramal do Incra	Nov 2016	1	106
P3	AM	374	Itacoatiara	Ramal do Minério	Nov 2016	11	333
P4	AM	374	Itacoatiara	Ramal do Minério	Nov 2016	11	333
P5	AM	93	Itacoatiara	Estr. Vila de Novo Remanso I	Nov 2016	0	268
P6	AM	93	Itacoatiara	Estr. Vila de Novo Remanso I	Nov 2016	0	268

* AC* Acre state,* AM* Amazonas state, *PA *Pará state,* RO* Rondônia state

^a^ID_SIVEP: SIVEP location. SIVEP: database of the Brazilian governmental program Sistema de Informação de Vigilância Epidemiológica da Malária (SIVEP-Malaria) that provides epidemiological and surveillance iInformation, with registration of all information and compulsory reporting of detected cases of malaria by all medical units and medical practitioners

^b^Number of cases of malaria in the month of collection and the previous month (Source: SIVEP-Malaria)

^c^Number of human inhabitants in the year the collection was performed (Source: SIVEP-Malaria)

Specimens were identified to species level using the morphological key of Forattini [36], then labeled and stored individually in silica gel at room temperature for subsequent analysis. Specimens from species complexes were molecularly identified using a 658-bp fragment of the barcode region of the mitochondrial cytochrome *c* oxidase subunit I gene (*COI*) using the protocol described in Bourke et al. [37]. The taxonomic nomenclature adopted in this study is that proposed by Foster et al. [38].

### Natural infectivity

Genomic DNA was extracted from all Anophelinae female mosquitoes using the Qiagen DNeasy Blood & Tissue Kit (Hilden, Germany). All DNA samples were tested for *Plasmodium* spp. infection as described by Sallum et al. [32]. DNA pools of up to five females containing equal amounts of genomic DNA (gDNA) were tested initially. Samples with a DNA concentration of < 1.0 ng/µl or > 15 ng/µl were tested individually and not pooled. Each real-time PCR was carried out in a final volume of 20 μl containing 1× PerfeCTa qPCR ToughMix, uracil *N*-glycosylase (UNG), ROX (Quanta BioSciences Inc., Gaithersburg, MD, USA), 0.3 μM each primer [39], ultrapure H_2_O and 2 μl of genomic DNA. The PCR thermal conditions were as follows: 5 min of UNG activation by holding at 45 °C; denaturation at 95 ºC/10 min, followed by amplification at 95 °C/15 s, 60 °C/1 min for 50 cycles. When the species of *Plasmodium* could not be detected with the triplex assay, conventional PCR was performed using primer pairs for *P. vivax* and *P. falciparum* [40].

### Malaria cases

The numbers of malaria cases and human population at risk by epidemiological week and annual parasite incidence (API) for both *P. vivax* and *P. falciparum* were requested from the Ministry of Health, Sistemas de Informações de Vigilância Epidemiológica (SIVEP)-Malaria [41], through the Electronic System of the Citizen Information Service (Sistema Eletrônico do Serviço de Informações ao Cidadão [SIC]) (https://esic.cgu.gov.br/sistema/site/index.aspx), protocols #25,820,001,316,201,742, #25,820,003,892,201,813, #25,820,004,426,201,847 and #25,820,004,717,201,835) (Table 1).

### Landscape analysis

Deforestation was measured as the amount of forest cover (FC), the sum of forest edges (i.e. a proxy for forest fragmentation, edge density [ED]) and the distance of the house from the drainage network (DW) in each location sampled. For each collection location, the percentage of FC in a 1-km radius (i.e. 3.14-km^2^ landscape) around the HLC house was calculated (see details in [32]). FC and other landscape metrics were calculated using European Space Agency (ESA) Sentinel 2A satellite imagery from the closest possible date to the field collection, minimizing cloud coverage. Radiometric and atmospheric corrections were performed using ESA’s Sen2Cor software v2.4.0 [42]. Maximum likelihood supervised classification was used to assign each pixel as forest or non-forest using spectral bands 2, 3 and 4. The percentage FC was calculated for each collection site as described in Prussing et al. [35]. The ED is a measure of the total perimeter of the edge of each remaining fragment of forest present within the same area used to calculated percentage FC. The DW was built from digital elevation models (DEMs) available on the ESA website, as well as from the satellite images used for classification. Thus, through processes in a geographic information system (GIS) environment, it was possible to design the DW for the collection areas. The DW was used to calculate the distance from each HLC collection point to the nearest body of water. All landscape metrics were calculated from circular images of a 1-km radius centered on the house in which the HLC collection was performed. For each of the 42 locations, a circular image and the three above-mentioned metrics were obtained. There was no overlap between locations sampled.

### Association between landscape, malaria cases and mosquitoes

Negative binomial regression as follows:

$$\ln \left( {\mu _{i} } \right) = \beta _{0} + \beta _{1} {\text{FC}}_{i} + \beta _{2} {\text{ED}}_{i} + \beta _{3} {\text{DW}}_{i} + \in$$

was used to estimate the association between each response variable: the number of local malaria cases, the number of *Ny. darlingi* in the peridomestic habitat, HBR and the number of *Plasmodium*-infected Anophelinae in the peridomestic habitat and the independent variables FC, ED and DW.

As response variables: the number of *Ny. darlingi* corresponded to the sum of specimens collected by HLC from 18:00 to 00:00 h in each peridomestic habitat. The number of infected mosquitoes refers to the total of anophelines positive for *Plasmodium* spp. in each peridomestic habitat and collected by HLC from 18:00 to 00:00 h. Data on total local malaria cases of *P. falciparum* and *P. vivax* registered in each locality sampled in the previous month and the collection months were gathered from the national surveillance system (SIVEP) (Table 1). HBR was employed as a proxy for the HLC rate because we assumed that the females collected were seeking a blood meal. Therefore, HBR is the total of *Ny. darlingi* collected in each peridomestic habitat per night (18:00–00:00 h) by one person. The independent variables were grouped in categories as shown in Table 2.

**Table 2:** Values that were considered to categorize explanatory variables in the negative binomial regression

Explanatory variables	Exposure	Values
FC		
1	Yes	30–70%
0	No	0–30% or 70–100%
ED		
1	Yes	0.0149 to 0.0292 (3rd and fourth quartiles, respectively)
0	No	0.0077 to 0.0141 (first and second quartiles, respectively)
DW		
1	Yes	≤ 138 m (median)
0	No	> 138 m (median)

*DW* Distance from the house at which human landing catch (HLC) collection was conducted to the nearest standing water, *ED* edge density, *FC* forest cover

The exponential of *β* represents the relative risk (IRR). We tested the following null hypothesis (H_0_: IRR = 1) with its alternative (H_a_: IRR ≠ 1) considering a significance level of 0.05 (type-I error or* α*) and confidence intervals (CIs) of (1 − *α*)%. An IRR > 1 indicates a reciprocal association between the response and independent variables, whereas IRR < 1 indicates that this relationship was non-reciprocal. Lastly, IRR = 1 indicates no association (null effect).

### Peak biting time

To determine the frequency and the peak biting time of *Plasmodium*-infected Anophelinae and *Ny. darlingi* the negative binomial regression was used as follows:

$$\ln \left( {\mu _{i} } \right) = \beta _{0} + \beta _{1} {\text{TI}}_{{1i}} + \beta _{2} {\text{TI}}_{{2i}} + \beta _{3} {\text{TI}}_{{3i}} + \beta _{4} {\text{TI}}_{{4i}} \; + \; \in$$

where the response variables were number of *Ny. darlingi* and number of *Plasmodium*-infected Anophelinae species. Data from 12-h collections in peridomestic habitats by HLC were used. The independent variable was the time interval (TI), stratified into quartiles. Each quartile corresponded to a 3-h time interval: first quartile, 18:00–21:00 h (baseline); second quartile, 21:00–00:00 h; third quartile, 00:00–03:00 h; and fourth quartile, 03:00–06:00 h. Analyses were performed by employing data from Acrelândia, Mâncio Lima and Cruzeiro do Sul, all in western Acre state, because 12-h collections were conducted only in these municipalities. We calculated the IRR and tested the hypothesis as described above.

### Infected mosquitoes and landscape and malaria

Binomial logistic regression was applied to estimate the relationship between the presence of infected mosquitoes in peridomestic habitats and landscape metrics and local malaria cases. Explanatory variables are shown in Table 3. This model was adjusted by the number of local malaria cases, which was categorized based on median value. For this analysis, data from 39 peridomestic habitats were used, rather than 42, because anopheline mosquitoes were not found in three habitats. All analyses were performed using Stata/IC 16.1 software [43].

**Table 3:** Values considered to categorize explanatory variables in binomial logistic regression

Explanatory variables	Exposure	Values
FC		
1	Yes	30–70%
0	No	0–30% or 70–100%
ED		
1	Yes	0.0150 to 0.0292
0	No	0.0077 to 0.0149
DW		
1	Yes	≤ 138 m (third and fourth quartiles)
0	No	> 138 m (first and second quartiles)
Number of local malaria cases		
1	Yes	> 13 (median)
0	No	≤ 13 (median)

## Results

### Mosquito collection and species identification

A total of 6962 anophelines of 40 species (Additional file 2: Table S1) were collected in peridomestic and forest edge habitats in localities with active malaria transmission. The ST collections at the forest edge comprised 1492 anopheline mosquitoes. Peridomestic collections accounted for 5470 Anophelinae mosquitoes, of which 4369 were collected by HLC and 1101 by BSS. In the peridomestic habitats (Additional file 2: Table S1), 90.22% of the collected Anophelinae were *Ny. darlingi*, 2.03% were *Ny. oryzalimnetes* Wilkerson & Motoki, 2009 and 1.86% were *Ny. braziliensis* (Chagas, 1907). The remaining species accounted for 5.89% of the collection. In the forest edge, 27.35% were *Ny. konderi* B, 24.33% were *Ny. triannulatus* (*s.l.*) (Neiva & Pinto, 1922) and 21.45% were *Ny. darlingi*. The remaining Anophelinae species accounted for 26.87% of the collection. Thirteen specimens of the genus *Chagasia* Cruz, 1906 were collected at the forest edge but none were tested for *Plasmodium.*

### Natural infectivity

A total of 6949 anopheline females were tested for *Plasmodium* infection, of which 69 were *P. vivax* positive, 20 were *P. falciparum* positive and two were *P. vivax* + *P. falciparum* positive (Table 4). The overall *Plasmodium* infection rate was 1.31%. *Plasmodium vivax* was identified in females collected in Mâncio Lima, Lábrea, Cruzeiro do Sul, Machadinho D’Oeste, Itacoatiara, Acrelândia and Pacajá, whereas *Ny. darlingi* infected with *P. falciparum* were collected in Mâncio Lima, Lábrea and Machadinho D’Oeste. *Nyssorhynchus darlingi* specimens with mixed *P. vivax* and *P. falciparum* infections were collected by HLC in a peridomestic habitat in Mâncio Lima. At the forest edge habitat, one *Ny. darlingi* collected in Lábrea, Amazonas state, was infected with *P*. *falciparum* and one *Ny*. *konderi* B from Acrelândia, Acre state had mixed *P*. *falciparum* and *P*. *vivax* infections. Most *Plasmodium*-infected mosquitoes were collected by HLC (*n* = 72), 17 by BSS; two were caught in a ST (Table 4). The hourly distribution of Anophelinae-infected females in the peridomestic habitats of each municipaltiy is shown in Additional file 3: Figure S2 and Additional file 4: Figure S3.

**Table 4:** Species of mosquitoes infected by *Plasmodium* spp., number of infected mosquitoes by environment (peridomestic and forest edge) and collection method

*Nyssorhynchus* spp.	*Plasmodium vivax*			*Plasmodium falciparum*			*Plasmodium vivax* + *P. falciparum*		
	Peridomestic site		Forest edge	Peridomestic site		Forest edge	Peridomestic site		Forest edge
	HLC	BS	ST	HLC	BS	ST	HLC	BS	ST
*Ny. darlingi*	60	6	0	9	10	1	1	0	0
*Ny. rangeli*	1	0	0	0	0	0	0	0	0
*Ny. benarrochi* B	1	1	0	0	0	0	0	0	0
*Ny. konderi* B	0	0	0	0	0	0	0	0	1

*BS* Barrier screen, *HLC* human landing catch, *ST* Shannon trap

### Association between landscape, malaria cases and mosquitoes

The results of the negative binomial regression showed the following. First, the number of local malaria cases was positively associated with ED (Additional file 5: Table S2). The malaria IRR was 5.63-fold higher (IRR = 6.63, 95% CI 3.34—13.17, *P* < 0.001) in landscapes exposed to fragmentation (ED ranging from 0.0149 to 0.0292) than in those not exposed to fragmentation (ED ranging from 0.0077 to 0.0141). Secondly, the number of *Plasmodium*-infected mosquitoes was positively associated with FC and negatively associated with DW (Additional file 6: Table S3). The IRR of *Plasmodium*-infected mosquitoes was 2.99-fold higher (IRR = 3.99, 95% CI 1.08–14.79, *P* = 0.038) in moderately disturbed landscapes (FC ranging from 30 to 70%) than in both highly altered (FC ranging from 0 to 30%) and conserved landscapes (FC ranging from 70 to 100%). The IRR of collecting infected mosquitoes in peridomestic habitats ≤ 138 m distant from a DW was 0.22-fold higher (IRR = 0.22, 95% CI 0.06–0.76, *p* = 0.017) than that in habitats > 138 m distant from a DW. Thirdly, the number of *Ny. darlingi* and HBR had no association with any independent variable (*P* > 0.05).

### Peak biting time

The regression analyses showed that the number of *Ny. darlingi* collected per hour decreased as the time approached 06:00 h, decreasing from 551 collected from 18:00–21:00 h, to 505 collected from 21:00–00:00 h, 313 collected from 00:00–03:00 h and 127 collected from 03:00–06:00 h. Using the data from 18:00–21:00 h as baseline, the lowest number of *Ny. darlingi* collected was from 03:00–06:00 h (IRR = 0.23, 95% CI 0.12–0.44, *P* < 0.001) (Additional file 7: Table S4).

In addition, the results also showed that the number of infected mosquitoes peaked (Table 5) after midnight, as opposed to the early evening peak abundance of collected *Ny. darlingi*. The IRR of *Plasmodium*-infected mosquitoes was 600% greater in the overnight period (00:00–03:00 h) than during the time period when the peak number of *Ny. darlingi* were collected (18: 00–21:00 h) (IRR = 7.0, 95% CI 1.04–46.97, *P* = 0.045) (Table 5).

**Table 5:** Negative binomial regression analysis on the number of *Plasmodium*-infected mosquitoes captured during the different collection intervals

Time period (h)	Number of *Plasmodium*-infected mosquitoes	Incidence rate ratio	Standard error	*P* value	95% Confidence interval
18:00–21:00	3	1.00			
21:00–00:00	8	2.67	2.695	0.332	0.368–19.323
00:00–03:00	21	7.00	6.800	0.045*	1.043–46.986
03:00–06:00	9	3.00	3.011	0.274	0.420–21.447
_cons		0.06	0.044	0.000	0.120–0.258

*Significant difference at *P* < 0.05

### Infected mosquitoes, landscape and malaria

In Itacoatiara (Amazonas state), three peridomestic habitats were negative for Anophelinae and subsequently excluded from the binomial logistic regression analyses. The presence of *Plasmodium*-infected mosquitoes in the peridomestic habitats was negatively associated with DW (odds ratio [OR] = 0.09, 95% CI 0.01–0.60, *P* = 0.013) and positively associated with the number of local malaria cases (OR = 9.41, 95% CI 1.49–59.65, *P* = 0.017) (Additional file 8: Table S5). No significant association was found with FC and ED. The OR of the presence of *Plasmodium*-infected mosquitoes was 91% lower in sites nearest the DW (DW ≤ 138 m) than in sites more distant from the DW (DW > 138 m). The OR of *Plasmodium*-infected mosquitoes in the peridomestic habitats was 8.41-fold higher in sites where the number of malaria cases was > 13 than in sites with ≤ 13 malaria cases.

## Discussion

Anthropogenic change in natural environments is a major driver of deforestation, habitat fragmentation and ecological changes that usually favor the emergence, resurgence or proliferation of zoonotic pathogens, reservoir hosts and mosquito vectors [25, 44–46]. Changes in land use and freshwater distribution can lead to the increased abundance and dispersion of mosquito vectors and the propagation of the pathogens they can carry as intermediate reservoir hosts [23, 24]. In addition, poor socioeconomic conditions can increase human exposure to pathogens, thus causing surges in and spreading of vector-borne infections, including malaria [28].

The status of *Ny. darlingi* as a dominant vector of *P. vivax* and *P. falciparum* has been reinforced by the findings of this study carried out in rural settlements in the Brazilian Amazon basin. The importance of the exophilic population of *Ny*. *darlingi* in Amazonian Brazil is strengthened by the fact that one female collected in ST in the forest edge was infected with *P. falciparum* in Lábrea, Amazonas, and a second female infected by both *P. vivax* and *P. falciparum* was collected in a peridomestic site in Mâncio Lima. In nearby Amazonian Peru, *Ny. darlingi* collected in peridomestic sites using the HLC method have been detected to be infected with *P. falciparum* in numerous communities in the peri-Iquitos region and Mazan district [47–49].

The results of this investigation reveal that other species other than *Ny. darlingi* can be local vectors of *Plasmodium* in the rural settlements sampled. Specimens of *Ny. rangeli* and *Ny. benarrochi* B were found infected with *P. vivax* in peridomestic habitats, and *Ny. konderi* B collected in the forest edge was found to be infected by *P. vivax* and *P. falciparum*. The importance of *Ny. benarrochi* B as a local vector of *P. vivax* was shown by in an earlier investigation carried out in eastern Peru [50] and in Putumayo, Colombia [51]. In the present study, females of *Ny. benarrochi* B infected with *P. vivax* were captured in HLC in Pacajá, Pará state, suggesting a local role for this species in *Plasmodium* dispersion among settlers. *Nyssorhynchus benarrochi* is a species complex composed of *Ny*. *benarrochi* (*s.s.*), *Ny*. *benarrochi* B, *Nyssorhynchus benarrochi* G1 and *Nyssorhynchus benarrochi* G2 [37]. Whereas *Ny*. *benarrochi* (*s.s.*) was identified based on morphology only, the other three members of the complex were defined by DNA sequence data. Thus, further work is necessary to obtain further evidence to separate these species using both morphology and DNA data and to identify those species that are vectors of *Plasmodium*. According to Bourke et al. [37], *Ny*. *benarrochi* B is found in Cruzeiro do Sul, Mâncio Lima, western Acre state and Peru, *Ny*. *benarrochi* G1 occurs in Acrelândia, Acre state and *Ny*. *benarrochi* G2 occurs in Machadinho D’Oeste, Rondônia state. *Nyssorhynchus rangeli* is a local vector that usually occurs at low frequencies in endemic areas. Females infected with *Plasmodium* spp. have been reported in Amapá state [52], southern Colombia [53] and southern Peru [54], whereas in the present study, *Ny*. *rangeli* was found infected with *P. vivax* only in Acrelândia, Acre state. *Nyssorhynchus konderi* B belongs to the Oswaldoi-Konderi Complex [55]. Other species of this complex have been found naturally infected with *Plasmodium* infection, such as *Ny. oswaldoi* (*s.l.*) and *Ny. konderi* (*s.l.*) [52, 53, 56, 57]. In the present study, *Plasmodium*-infected *Ny. konderi* B was collected by ST at the forest edge in a location with approximately 84% forest cover, where *Ny. konderi* B was a dominant species (334 specimens of *Ny. konderi* B among 506 Culicidae collected).

The positive association between the number of local malaria cases and forest fragmentation, using ED as a proxy of fragmentation, and between the number of *Plasmodium*-infected anophelines and percentage of FC reinforce previous findings that forest clearing is a risk factor for malaria in Amazonian settlements [26, 58, 59]. Undisturbed areas of the Amazon forest do not provide favorable ecological and environmental conditions for the proliferation of *Ny. darlingi* [60]. In previous field collections carried out in reserves and undisturbed forest locations throughout Amazonas state, *Ny. darlingi* was either absent or rare [61–64], compared with sylvatic species of the *Stethomyia*, *Lophopodomia* and *Chagasia* genera of the subfamily Anophelinae. The loss and fragmentation of FC allow sunlight to reach the soil, leading to changes in freshwater conditions (e.g. in the temperature and pH, among other factors) and increasing the suitability of the larval habitat for *Ny. darlingi* [21, 60]. Together with local socioecological factors, a high density of vector species and high prevalence of infection in humans and in the vector populations, malaria transmission becomes endemic and sustainable [28]. Even though the conditions of many Amazonian rural settlements support malaria transmission, disease distribution remains heterogeneous. This uneven occurrence can be explained by rapid urbanization and improved socioeconomic conditions in consolidated settlements, whereas transmission is high in areas where the economic activity is linked to exploitation of forest products and agricultural settlements [8], associated with continuous deforestation [59]. The transmission intensity of *P. vivax* infection in some Amazonian rural settlements can be as high as that for *P. falciparum* in sub-Saharan Africa, whereas in other rural settlements the transmission is low [32, 34]. The risk of acquiring a vector-borne pathogen, including malaria linked to habitat fragmentation, decreases when the distribution of mosquitoes is proportional to that of humans [65].

The results of the analyses showed a negative association between the distance of the household to the nearest water DW and the presence and number of *Plasmodium*-infected anophelines in the peridomestic habitat, including *Ny. darlingi*. This finding indicates that residents of houses closer to a DW have a lower risk of being exposed to anopheline bites and *Plasmodium*-infected females—and contradicts previous findings that demonstrated that residents of houses closer to forest edges have a higher risk of acquiring malaria [58]. However, the DW is a landscape metric, not a proxy of the distance of a household from the forest edge. In addition, our findings can be understood in the context of the distribution of the primary larval habitat of *Ny. darlingi* in the sampled areas. Despite our lack of information on local larval habitat distribution, it is not uncommon to detect water redistribution linked to human land occupation for farming development and food production [23]. In rural areas of the Brazilian Amazon, freshwater redistribution has been found to increase the number of *Ny. darlingi* larval habitats and the density of this species in a human-dominated environment [21, 27]. Fish-farming ponds also result in the redistribution of freshwater and are associated with epidemic and endemic malaria transmission in urban, periurban and rural areas in western Amazonia [66, 67], Peruvian Amazon [68] and western Kenya [69]. Both commercial and non-commercial pisciculture, particularly when either abandoned or situated at the edge of vegetation that is not cleared constantly, lead to higher mosquito abundance, including malaria vector species [35, 66, 67].

Heterogeneous biting behavior of *Ny. darlingi* in terms of blood-feeding inside and outside houses and variations in the peak time of biting have been shown by numerous studies carried out across the Amazon [60]. Although along the Napo River in Mazán District, Peru, *Ny. darlingi* bites outdoors more frequently than indoors [49], in San José de Lupuna and Cahuide in peri-Iquitos state, Peru, the mosquito showed a trend of increased feeding inside houses, but only after the repellent effects of long-lasting insecticide-treated nets were presumed to have worn off [48]. In addition, variation in biting behavior was reported in French Guiana, where *Ny. darlingi* peaks between 20:30 and 22:30 h [70], and in Venezuela, this mosquito bites throughout the night, with minor peaks at 23:00–00:00 h and 03:00–04:00 h [71]. In the northeastern Amazon, in Macapá municipality, Amapá state, *Ny. darlingi* was detected with either a low but continuous biting peak [72] or multimodal biting peaks throughout the night [73]. Changes in biting behavior of *Ny. darlingi* can be associated with deforestation, ecological factors and microclimate conditions, which emerge due to changes in temperature and humidity. In Iquitos, Peruvian Amazon, the biting rate of this vector species was found to be significantly higher in a region that was undergoing massive deforestation associated with road construction than in locations where the forest remained mostly undisturbed [74].

In the present study, the 12-h HLC collections revealed that the peak time of biting of *Plasmodium*-infected Anophelinae was from 00:00 to 03:00 h in Mâncio Lima, Acrelândia and Cruzeiro do Sul, Acre state. In these regions, although infected mosquitoes were collected throughout the 12-h collections, 73% of the infected mosquitoes (30/41) were captured between 00:00 and 06:00 h. Results of previous studies have shown that the frequency of nulliparous females was higher from 18:00 to 22:00 h, whereas parous females were more frequently collected from 02:00 to 06:00 h [75]. With respect to the latter, the likelihood of collecting infected females in our study should be higher in the second period. The peak biting time of *Plasmodium*-infected females may also be linked to a daily rhythmic behavior that protects females from desiccation by flying at night, increases female longevity and increases biting success and capacity to transmit sporozoites to susceptible hosts at a time when they are less defensive [76]. An experimental study employing *P. chabaudi* genotype AS parasites and *Anopheles stephensi* showed that the interaction between increased gametocyte infectiousness at night and increased mosquito susceptibility to infection enhanced parasite transmission [77]. A similar adaptive periodicity and protective behavior from high temperature/low humidity may occur in mosquitoes that are involved in *Plasmodium* transmission to humans.

The results of this study underscore the importance of the dominant malaria vector, *Ny*. *darlingi*, and other vector species of human *Plasmodium* in the Brazilian Amazon. The involvement of several Anophelinae species in the dynamics of the *Plasmodium* spp. transmission cycle adds complexity to parasite–vector associations, with implications for an effective malaria control in a region that being impacted by extensive anthropogenic disturbances in the forest environment. The delineation of interventions for malaria control in a heterogeneous scenario of transmission that involves distinct species will require continuous investigation, primarily to verify the role of each species in transmission. Investigations focusing on the knowledge of field malariology are required to define the local determinants of malaria transmission, as denoted by Baird [78] and Benelli and Beier [79]. Ecological characteristics, such as variation in mosquito behavior, vector biodiversity, lack of knowledge of the impact of environmental change on mosquito ecology, among others, can impede the success of malaria control and elimination programs [79].

## Conclusions

*Nyssorhynchus benarrochi* B and *Ny. konderi* B were found to be naturally infected with *Plasmodium*, and our results corroborate the importance of *Ny. rangeli* as a secondary vector of *Plasmodium*, adding complexity to a program targeting the control of multiple vectors. Forest fragmentation favored an increase in malaria occurrence. The likelihood of being exposed to *Plasmodium*-infected mosquitoes in the peridomestic environment is lower in households situated closer to a drainage network than in more distantly located houses. The early hours of the morning were found to present greater risk for being bitten by an infected female in the settlements studied. Thus, the findings of this study provide novel ecological knowledge about anopheline vectors of *Plasmodium* in rural settlements in the Amazon basin and reinforce the importance of the use of impregnated mosquito nets and screens in windows and doors. Further studies should be carried out to verify if the period of higher biting activity described here is also found in landscapes of other rural settlements and to determine the factors contributing to this pattern.

## Supplementary Information

**Additional file 1. Figure S1.** Drawing illustrating the peridomestic and forest edge habitats where the barrier screen and Shannon trap mosquito collections were carried out.

**Additional file 2. Table S1.** Species of the subfamily Anophelinae collected in peri-domestic and forest edge habitats in rural settlements across the Brazilian Amazon.

**Additional file 3. Figure S2.** Hourly distribution and number of Anophelinae and *Plasmodium*-infected mosquitoes in 12 h collections in peridomestic habitat. Collections of 12 h were performed in Cruzeiro do Sul, Acrelândia and Mâncio Lima municipalities, Acre state, Brazil.

**Additional file 4. Figure S3.** Hourly distribution and number of Anophelinae and *Plasmodium*-infected mosquitoes in 6-h collections in peridomestic habitat. Collections of 6 h were performed in Lábrea (Acre state), Itacoatiara (Amazonas state), Machadinho D’Oeste (Rondônia state), Pacajá (Pará state) municipalities, Brazil. Note differences in* y*-axis scales.

**Additional file 5. Table S2.** Final model of the negative binomial regression analysis for the response variable: number of local malaria cases.

**Additional file 6. Table S3.** Final multiple models of the negative binomial regression analysis for the response variable: number of infected mosquitoes.

**Additional file 7. Table S4.** Negative binomial regression analysis. Number of *Ny. darlingi* in the different collection intervals.

**Additional file 8. Table S5.** Final multiple models of the binomial logistic regression analysis.

## Data Availability

Data used in the manuscript will be freely available to any scientist wishing to use them for non-commercial purposes upon request to the corresponding author. In addition, the full data will be publicly available after publication of a series of articles in preparation.

## Abbreviations

API: Annual parasite incidence
BSS: Barrier screen sampling
COI: Cytochrome *c* oxidase subunit I gene
C_4_H_8_O_2_: Ethyl acetate
DEM: Digital elevation model
DW: Drainage network
ED: Edge density
ESA: European Space Agency
FC: Forest cover
GIS: Geographic information system
GML: Generalized linear model
HBR: Human biting rate
HLC: Human landing catch
ICMBio: Instituto Chico Mendes de Conservação da Biodiversidade
IRR: Incidence ratio rate
SIC: Serviço de Informações ao Cidadão
SIVEP: Sistemas de Informações de Vigilância Epidemiológica
ST: Shannon trap
