# Supplementary material for: Vector role and human biting activity of Anophelinae mosquitoes in different landscapes in the Brazilian Amazon
Source: Parasit Vectors. 2021 May 6;14:236. doi: 10.1186/s13071-021-04725-2 (PMC8101188; doi:10.1186/s13071-021-04725-2)
Supplement: Supplementary file 2 — Additional file 2. Table S1. Species of the subfamily Anophelinae collected in peri-domestic and forest edge habitats in rural settlements across the Brazilian Amazon. [file 13071_2021_4725_MOESM2_ESM.docx]

**Vector role and human biting activity of Anophelinae mosquitoes in different landscapes in the Brazilian Amazon**

Tatiane M. P. de Oliveira^1^, Gabriel Z. Laporta^2^, Eduardo S. Bergo^3^, Leonardo Suveges Moreira Chaves^1^, José Leopoldo F. Antunes^1^, Sara A. Bickersmith^4^, Jan E. Conn^4,5^, Eduardo Massad^6^, Maria AniceMureb Sallum^1#^

^1^Departamento de Epidemiologia, Faculdade de Saúde Pública, Universidade de São Paulo, São Paulo, SP, BR.

^2^Setor de Pós-graduação, Pesquisa e Inovação, Centro Universitário Saúde ABC, (FMABC) Fundação ABC, Santo André, SP, BR.

^3^Superintendencia de Controle de Endemias, Secretaria de Estado da Saúde, SP, BR.

^4^Wadsworth Center, New York State Department of Health, Albany, NY, USA.

^5^Department of Biomedical Sciences, School of Public Health, State University of New York, Albany, NY, USA.

^6^Matemática Aplicada, Fundação Getulio Vargas, Rio de Janeiro, RJ, BR

Author’s email:

Tatiane M. P. Oliveira: porangaba@usp.br

Gabriel Z. Laporta: gabriel.laporta@fmabc.br

Eduardo Bergo: edusteber@uol.com.br

Leonardo Chaves: leonardosuveges@usp.br

José Leopoldo F. Antunes: leopoldo@usp.br

Sara A. Bickersmith: sara.bickersmith@health.ny.gov

Jan E. Conn: jan.conn@health.ny.gov

Eduardo Massad: edmassad@dim.fm.usp.br

Maria A. M. Sallum: masallum@usp.br

^#^Corresponding author:

Tatiane M. P. de Oliveira. Faculdade de Saúde Pública. Av. Dr. Arnaldo, 715, Cerqueira César. São Paulo, SP, CEP 01246-904.

| **Additional file 2. Table S1.** Species of the subfamily Anophelinae collected in peri-domestic and forest edge habitats in rural settlements across the Brazilian Amazon. | | | | | |
| --- | --- | --- | --- | --- | --- |
| **Species** | **Peridomestic** | **Forest edge** |  |  |  |
| *An. costai* | absent | present |  |  |  |
| *An.* nearc*ostai* | absent | present |  |  |  |
| *An. costai* G1 | present | present |  |  |  |
| *An. costai* G2 | present | absent |  |  |  |
| *An. costai* G3 | absent | present |  |  |  |
| *An. costai* G4 | present | present |  |  |  |
| *An.* near*fluminensis* | absent | present |  |  |  |
| *An.*near*fluminensis*G1 | absent | present |  |  |  |
| *An.* near*fluminensis*G2 | absent | present |  |  |  |
| *An.* near*fluminensis*G3 | absent | present |  |  |  |
| *An. nearmalefactor* | absent | present |  |  |  |
| *An. matogrossensis* | present | present |  |  |  |
| *An. minor* | absent | present |  |  |  |
| *An. peryassui* | present | present |  |  |  |
| *An. punctimacula* | present | present |  |  |  |
| *An.* near*punctimacula* | absent | present |  |  |  |
| *Chagasiafajardi* | present | present |  |  |  |
| *Chagasia*sp. | absent | present |  |  |  |
| *Ny. albitarsis* | present | absent |  |  |  |
| *Ny. albitarsis*s.l. | present | absent |  |  |  |
| *Ny. arthuri* C | present | absent |  |  |  |
| *Ny. benarrochi*B | present | present |  |  |  |
| *Ny. braziliensis* | present | present |  |  |  |
| *Ny. darlingi* | present | present |  |  |  |
| *Ny. deaneorum* | present | present |  |  |  |
| *Ny. dunhami* | absent | present |  |  |  |
| *Ny. galvaoi* | present | absent |  |  |  |
| *Ny. goeldii* | present | present |  |  |  |
| *Ny. konderi*s.l. | present | present |  |  |  |
| *Nykonderi*A | absent | present |  |  |  |
| *Ny. konderi* B | present | present |  |  |  |
| *Ny. nuneztovari*s.l. | present | absent |  |  |  |
| *Ny. oryzalimnetes* | present | present |  |  |  |
| *Ny. oswaldoi* | absent | present |  |  |  |
| *Ny. oswaldoi*s.s. | absent | present |  |  |  |
| *Ny. oswaldoi*s.l. | absent | present |  |  |  |
| *Ny. oswaldoi*A | present | present |  |  |  |
| *Ny. rangeli* | present | present |  |  |  |
| *Ny. triannulatus* | present | present |  |  |  |
| *Stethomyia*sp. | absent | present |  |  |  |
