# Supplementary material for: Vector role and human biting activity of Anophelinae mosquitoes in different landscapes in the Brazilian Amazon
Source: Parasit Vectors. 2021 May 6;14:236. doi: 10.1186/s13071-021-04725-2 (PMC8101188; doi:10.1186/s13071-021-04725-2)
Supplement: Supplementary file 4 — Additional file 4. Figure S3. Hourly distribution and number of Anophelinae and Plasmodium-infected mosquitoes in 6-h collections in peridomestic habitat. Collections of 6 h were performed in Lábrea (Acre state), Itacoatiara (Amazonas state), Machadinho D’Oeste (Rondônia state), Pacajá (Pará state) municipalities, Brazil. Note differences in y-axis scales. [file 13071_2021_4725_MOESM4_ESM.docx]

**Vector role and human biting activity of Anophelinae mosquitoes in different landscapes in the Brazilian Amazon**

Tatiane M. P. de Oliveira^1^, Gabriel Z. Laporta^2^, Eduardo S. Bergo^3^, Leonardo Suveges Moreira Chaves^1^, José Leopoldo F. Antunes^1^, Sara A. Bickersmith^4^, Jan E. Conn^4,5^, Eduardo Massad^6^, Maria AniceMureb Sallum^1#^

^1^Departamento de Epidemiologia, Faculdade de Saúde Pública, Universidade de São Paulo, São Paulo, SP, BR.

^2^Setor de Pós-graduação, Pesquisa e Inovação, Centro Universitário Saúde ABC, (FMABC) Fundação ABC, Santo André, SP, BR.

^3^Superintendencia de Controle de Endemias, Secretaria de Estado da Saúde, SP, BR.

^4^Wadsworth Center, New York State Department of Health, Albany, NY, USA.

^5^Department of Biomedical Sciences, School of Public Health, State University of New York, Albany, NY, USA.

^6^Matemática Aplicada, Fundação Getulio Vargas, Rio de Janeiro, RJ, BR

Author’s email:

Tatiane M. P. Oliveira: porangaba@usp.br

Gabriel Z. Laporta: gabriel.laporta@fmabc.br

Eduardo Bergo: edusteber@uol.com.br

Leonardo Chaves: leonardosuveges@usp.br

José Leopoldo F. Antunes: leopoldo@usp.br

Sara A. Bickersmith: sara.bickersmith@health.ny.gov

Jan E. Conn: jan.conn@health.ny.gov

Eduardo Massad: edmassad@dim.fm.usp.br

Maria A. M. Sallum: masallum@usp.br

^#^Corresponding author:

Tatiane M. P. de Oliveira. Faculdade de Saúde Pública. Av. Dr. Arnaldo, 715, Cerqueira César. São Paulo, SP, CEP 01246-904.


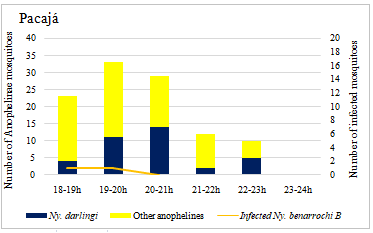

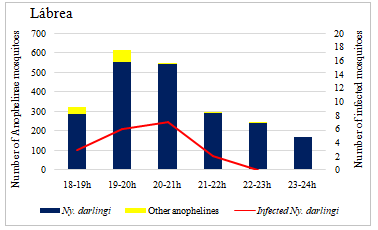

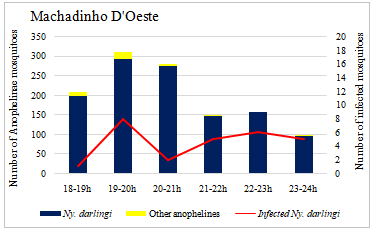

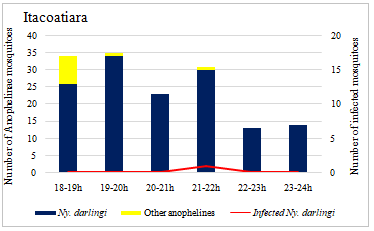


**Additional file 4. Figure S3.** Hourly distribution and number of Anophelinae and *Plasmodium*-infected mosquitoes in 6 h collections in peridomestic habitats. Collections of 6 h were performed in Lábrea (Acre state), Itacoatiara (Amazonas state), Machadinho D’Oeste (Rondônia state), Pacajá (Pará state) municipalities, Brazil. Note differences in y-axis scales.
