# Supplementary material for: Vector role and human biting activity of Anophelinae mosquitoes in different landscapes in the Brazilian Amazon
Source: Parasit Vectors. 2021 May 6;14:236. doi: 10.1186/s13071-021-04725-2 (PMC8101188; doi:10.1186/s13071-021-04725-2)
Supplement: Supplementary file 6 — Additional file 6. Table S3. Final multiple models of the negative binomial regression analysis for the response variable: number of infected mosquitoes. [file 13071_2021_4725_MOESM6_ESM.docx]

**Vector role and human biting activity of Anophelinae mosquitoes in different landscapes in the Brazilian Amazon**

Tatiane M. P. de Oliveira^1^, Gabriel Z. Laporta^2^, Eduardo S. Bergo^3^, Leonardo Suveges Moreira Chaves^1^, José Leopoldo F. Antunes^1^, Sara A. Bickersmith^4^, Jan E. Conn^4,5^, Eduardo Massad^6^, Maria AniceMureb Sallum^1#^

^1^Departamento de Epidemiologia, Faculdade de Saúde Pública, Universidade de São Paulo, São Paulo, SP, BR.

^2^Setor de Pós-graduação, Pesquisa e Inovação, Centro Universitário Saúde ABC, (FMABC) Fundação ABC, Santo André, SP, BR.

^3^Superintendencia de Controle de Endemias, Secretaria de Estado da Saúde, SP, BR.

^4^Wadsworth Center, New York State Department of Health, Albany, NY, USA.

^5^Department of Biomedical Sciences, School of Public Health, State University of New York, Albany, NY, USA.

^6^Matemática Aplicada, Fundação Getulio Vargas, Rio de Janeiro, RJ, BR

Author’s email:

Tatiane M. P. Oliveira: porangaba@usp.br

Gabriel Z. Laporta: gabriel.laporta@fmabc.br

Eduardo Bergo: edusteber@uol.com.br

Leonardo Chaves: leonardosuveges@usp.br

José Leopoldo F. Antunes: leopoldo@usp.br

Sara A. Bickersmith: sara.bickersmith@health.ny.gov

Jan E. Conn: jan.conn@health.ny.gov

Eduardo Massad: edmassad@dim.fm.usp.br

Maria A. M. Sallum: masallum@usp.br

^#^Corresponding author:

Tatiane M. P. de Oliveira. Faculdade de Saúde Pública. Av. Dr. Arnaldo, 715, Cerqueira César. São Paulo, SP, CEP 01246-904.

**Additional file 6.Table S3.** Final multiple models of the negative binomial regression analysis for the response variable: number of infected mosquitoes.

| Number of infected mosquitoes | IRR | Std. Err. | z | *p*value | 95% Conf. interval |
| --- | --- | --- | --- | --- | --- |
| DW | 0.22 | 0.139 | -2.38 | 0.017* | 0.062 – 0.764 |
| FC | 3.99 | 2.667 | 2.07 | 0.038* | 1.077 – 14.790 |
| _cons | 0.68 | 0.391 | -0.68 | 0.498 | 0.217 – 2.099 |

DW: distance from human landing catch houses to the nearest standing water; FC: forest cover; IRR: incidence rate ratios; _cons: constant.

*Significance level (*p*) < 0.05
