# Supplementary material for: Vector role and human biting activity of Anophelinae mosquitoes in different landscapes in the Brazilian Amazon
Source: Parasit Vectors. 2021 May 6;14:236. doi: 10.1186/s13071-021-04725-2 (PMC8101188; doi:10.1186/s13071-021-04725-2)
Supplement: Supplementary file 7 — Additional file 7. Table S4. Negative binomial regression analysis. Number of Ny. darlingi in the different collection intervals. [file 13071_2021_4725_MOESM7_ESM.docx]

**Vector role and human biting activity of Anophelinae mosquitoes in different landscapes in the Brazilian Amazon**

Tatiane M. P. de Oliveira^1^, Gabriel Z. Laporta^2^, Eduardo S. Bergo^3^, Leonardo Suveges Moreira Chaves^1^, José Leopoldo F. Antunes^1^, Sara A. Bickersmith^4^, Jan E. Conn^4,5^, Eduardo Massad^6^, Maria AniceMureb Sallum^1#^

^1^Departamento de Epidemiologia, Faculdade de Saúde Pública, Universidade de São Paulo, São Paulo, SP, BR.

^2^Setor de Pós-graduação, Pesquisa e Inovação, Centro Universitário Saúde ABC, (FMABC) Fundação ABC, Santo André, SP, BR.

^3^Superintendencia de Controle de Endemias, Secretaria de Estado da Saúde, SP, BR.

^4^Wadsworth Center, New York State Department of Health, Albany, NY, USA.

^5^Department of Biomedical Sciences, School of Public Health, State University of New York, Albany, NY, USA.

^6^Matemática Aplicada, Fundação Getulio Vargas, Rio de Janeiro, RJ, BR

Author’s email:

Tatiane M. P. Oliveira: porangaba@usp.br

Gabriel Z. Laporta: gabriel.laporta@fmabc.br

Eduardo Bergo: edusteber@uol.com.br

Leonardo Chaves: leonardosuveges@usp.br

José Leopoldo F. Antunes: leopoldo@usp.br

Sara A. Bickersmith: sara.bickersmith@health.ny.gov

Jan E. Conn: jan.conn@health.ny.gov

Eduardo Massad: edmassad@dim.fm.usp.br

Maria A. M. Sallum: masallum@usp.br

^#^Corresponding author:

Tatiane M. P. de Oliveira. Faculdade de Saúde Pública. Av. Dr. Arnaldo, 715, Cerqueira César. São Paulo, SP, CEP 01246-904.

| **Additional file 7.Table S4.** Negative binomial regression analysis. Number of *Ny. darlingi* in the different collection intervals.   \| Time Timeperiod (h) \| IRR \| Std. Err. \| *p*value \| 95% Conf. Interval \| \| \| --- \| --- \| --- \| --- \| --- \| --- \| \| 18:00 – 21:00 \| 1.00 \|  \|  \| \|  \| \| \| 21:00 – 00:00 \| 0.92 \| 0.295 \| 0.787 \| \| 0.487 – 1.723 \| \| \| 00:00 – 03:00 \| 0.57 \| 0.184 \| 0.081 \| \| 0.300 – 1.073 \| \| \| 03:00 – 06:00 \| 0.23 \| 0.076 \| 0.000* \| \| 0.120 – 0.441 \| \| \| _cons \| 10.20 \| 2.323 \| 0.000 \| \| 6.531 – 15.941 \| \| |
| --- | --- | --- | --- | --- | --- | --- | --- | --- | --- | --- | --- | --- | --- | --- | --- | --- | --- | --- | --- | --- | --- | --- | --- | --- | --- | --- | --- | --- | --- | --- | --- | --- | --- | --- | --- | --- | --- | --- | --- | --- | --- |

IRR: incidence rate ratios; _cons: constant.

*Significance level (*p*) < 0.05
