# Supplementary material for: Vector role and human biting activity of Anophelinae mosquitoes in different landscapes in the Brazilian Amazon
Source: Parasit Vectors. 2021 May 6;14:236. doi: 10.1186/s13071-021-04725-2 (PMC8101188; doi:10.1186/s13071-021-04725-2)
Supplement: Supplementary file 8 — Additional file 8. Table S5. Final multiple models of the binomial logistic regression analysis. [file 13071_2021_4725_MOESM8_ESM.docx]

**Vector role and human biting activity of Anophelinae mosquitoes in different landscapes in the Brazilian Amazon**

Tatiane M. P. de Oliveira^1^, Gabriel Z. Laporta^2^, Eduardo S. Bergo^3^, Leonardo Suveges Moreira Chaves^1^, José Leopoldo F. Antunes^1^, Sara A. Bickersmith^4^, Jan E. Conn^4,5^, Eduardo Massad^6^, Maria AniceMureb Sallum^1#^

^1^Departamento de Epidemiologia, Faculdade de Saúde Pública, Universidade de São Paulo, São Paulo, SP, BR.

^2^Setor de Pós-graduação, Pesquisa e Inovação, Centro Universitário Saúde ABC, (FMABC) Fundação ABC, Santo André, SP, BR.

^3^Superintendencia de Controle de Endemias, Secretaria de Estado da Saúde, SP, BR.

^4^Wadsworth Center, New York State Department of Health, Albany, NY, USA.

^5^Department of Biomedical Sciences, School of Public Health, State University of New York, Albany, NY, USA.

^6^Matemática Aplicada, Fundação Getulio Vargas, Rio de Janeiro, RJ, BR

Author’s email:

Tatiane M. P. Oliveira: porangaba@usp.br

Gabriel Z. Laporta: gabriel.laporta@fmabc.br

Eduardo Bergo: edusteber@uol.com.br

Leonardo Chaves: leonardosuveges@usp.br

José Leopoldo F. Antunes: leopoldo@usp.br

Sara A. Bickersmith: sara.bickersmith@health.ny.gov

Jan E. Conn: jan.conn@health.ny.gov

Eduardo Massad: edmassad@dim.fm.usp.br

Maria A. M. Sallum: masallum@usp.br

^#^Corresponding author:

Tatiane M. P. de Oliveira. Faculdade de Saúde Pública. Av. Dr. Arnaldo, 715, Cerqueira César. São Paulo, SP, CEP 01246-904.

**Additional file 8.Table S5.**  Final multiple models of the binomial logistic regression analysis.

| **Presence/absence of**  **infected mosquitoes** | **Odds Ratio** | **Std. Err.** | **z** | ***p*value** | **95% Conf. interval** |
| --- | --- | --- | --- | --- | --- |
| **FC** | 4.62 | 4.112 | 1.72 | 0.085 | 0.809 – 26.430 |
| **DW** | 0.09 | 0.088 | -2.49 | 0.013* | 0.014 – 0.604 |
| **Local malaria cases** | 9.41 | 8.867 | 2.38 | 0.017* | 1.485 – 59.649 |
| **_cons** | 0.26 | 0.215 | -1.63 | 0.104 | 0.050 – 1.322 |

FC: forest cover, DW: distance from human landing catch houses to the nearest standing water; _cons: constant.

*Significance level (*p*) < 0.05
